# Safety of Commercial Cod Products and Potential Impact on Consumers

**DOI:** 10.3390/foods15071202

**Published:** 2026-04-02

**Authors:** Vincenzo Nava, Angela Giorgia Potortì, Vincenzo Lo Turco, Irene Maria Spanò, Roberto Sturniolo, Giuseppa Di Bella

**Affiliations:** Department of Biomedical, Dental and Morphological and Functional Imaging Sciences (BIOMORF), University of Messina, Viale Palatucci 13, 98168 Messina, Italy; vnava@unime.it (V.N.); agpotorti@unime.it (A.G.P.); irene.spano@studenti.unime.it (I.M.S.); roberto.sturniolo@studenti.unime.it (R.S.); gdibella@unime.it (G.D.B.)

**Keywords:** cod, inorganic elements, biogenic amines, quality, safety

## Abstract

This study examines the safety of commercial cod products by analysing the elemental profile and biogenic amine content of ten types of cod (fresh and processed) purchased in Messina and determining the intake of inorganic elements. The results demonstrate the influence of processing techniques on the content of essential and toxic elements, as well as biogenic amines, in processed products compared to fresh products. Histamine content was consistently below the permitted maximum. All species were found to be good sources of macronutrients (Ca, K, Mg, P and Na) and moderate sources of certain micronutrients (Fe, Zn, Cu and Se). However, some toxic elements (Cd and As) showed high concentrations, often exceeding reference limits. Assuming an average daily cod consumption of 100 g, some products exceeded the daily requirement for certain inorganic elements (As, Na, P, Se). Therefore, reducing the daily intake of these products would be advisable to mitigate potential toxicological risks. These findings help to define the safety profile of cod-based products, which are now commonly present in our diet. They also confirm the need for constant monitoring to ensure consumer protection in an ever-evolving market.

## 1. Introduction

Thanks to their particularly rich and varied nutritional profile, fish products have been a fundamental component of the human diet for centuries. They are an excellent source of high-quality proteins. Additionally, fish products contain omega-3 polyunsaturated fatty acids, including eicosapentaenoic (EPA) and docosahexaenoic (DHA) acids, which are renowned for their positive impact on cardiovascular and neurological health [1,2]. Moreover, this matrix is also important in terms of the content of vitamins and essential inorganic elements [3].

In addition to their nutritional value, fish products are renowned for their culinary versatility and their suitability for balanced eating patterns such as the Mediterranean diet, which recommends regular consumption [4]. However, contemporary eating habits are changing rapidly, as is the way we consume fish. Indeed, the growing global demand for fish products, coupled with consumers’ fast-paced lifestyles, has led to the development of a wide range of processed or ready-to-eat fish products in recent decades [3]. These include frozen fillets, canned products, semi-finished products, and ready-to-eat products. These products now represent an important part of the human diet [5]. This term generally refers to food products that have undergone processing, including washing, cutting and pre-cooking, and that are stored and distributed at room temperature, refrigerated or frozen, in suitable packaging [6].

The choice to consume ready-to-eat or processed fish products is primarily driven by convenience. By eliminating the cleaning, filleting and preparation stages, fish becomes more accessible, even for those who are short on time or lack culinary skills. Additionally, the availability of processed products enables consumers to enjoy fish species that would otherwise be difficult to find fresh or out of season, thereby increasing food variety. Another advantage of consuming these products is that they come in calibrated portions, reducing household waste. Furthermore, industrial processes should ensure high hygiene standards and greater food safety [7]. These processes not only prolong shelf life and maintain quality, but also eliminate most microorganisms [8].

However, alongside the advantages, there are also some critical aspects. Heat treatments and preservation techniques can lead to a partial loss of heat-sensitive nutrients, such as certain vitamins, or alter the quality of lipids [9,10]. For instance, the EPA and DHA content of fish can be significantly affected by cooking, since these nutrients are highly susceptible to oxidation [11]. Many ready-to-eat products also contain salt, preservatives or additives, which can negatively impact the overall quality of the diet if consumed frequently. From a sustainability perspective, the environmental impact of packaging and industrial processes must also be considered. Consequently, while these transformations guarantee a longer shelf life and greater ease of use, careful monitoring of nutritional and safety characteristics is required, since processing and storage can affect the concentration of bioactive compounds and the bioavailability of bioactive components [12]. Furthermore, environmental contamination necessitates constant monitoring of the quality of fish and fish products, particularly for species susceptible to bioaccumulation and biomagnification. Contamination can occur in the marine or river environment, as well as during post-harvest processes such as processing, storage and packaging, raising specific issues for fresh fish compared to processed products.

Traditionally processed fish products also pose a high risk to human health due to halophilic pathogenic bacteria and biogenic amines [13]. Biogenic amines (BAs) are a class of compounds derived from amino acids. Their production depends on the availability of amino acids and the synthesis of amino acid decarboxylases by bacteria [14]. Therefore, quantifying amines in fish is considered an important quality indicator and a valuable health surveillance tool [15]. Furthermore, the content of biogenic amines can vary depending on various factors, such as processing techniques, product properties (e.g., pH value, water activity (aw), and salt content), and other ingredients used [13]. Technological processes such as salting, ripening, fermentation and marinating can increase the likelihood of BAs forming. A pH of 4.0–5.5 is favourable for enhanced amino acid decarboxylase activity and can be achieved in salted anchovies, for instance [16].

However, the potential risk to humans posed by the presence of toxic and potentially toxic elements in fish products must also not be overlooked. Although several regulations govern the control and safety of these products, the risk of inorganic contamination remains a concern. These elements can contaminate fish products at any stage of post-harvest handling or processing, for example through contact with unsuitable surfaces or equipment such as countertops, knives or metal containers, or through the water used for washing or preservation and packaging materials [17]. It is important, however, not to overlook environmental factors that influence the level of contamination from toxic elements in fish products.

Cod is one of the most used species of fish in food processing [18]. Cod is native to the Atlantic Ocean, where *Gadus morhua* is the best-known and most historically widespread species. Over time, however, the *Gadidae* family has evolved into several taxonomic varieties that now inhabit other marine basins, including those analysed in this study (see Table 1). This fish has been a staple food and almost the only source of protein for human civilization for centuries. Since its earliest use, cod has been distinguished by its high nutrient content. Its ease of preservation is another factor in its popularity, as its low-fat flesh does not go rancid easily, unlike that of other fish. This means that, by avoiding bacterial contamination, cod flesh can be preserved for a long time. In the past, salt was one of the ingredients used to preserve cod, as it is a powerful antibacterial agent and allows the cells to dehydrate. It is no coincidence that salted cod is still so widespread, going by different names in different regions.

Cod is particularly popular in Italy, which is one of the main European markets for this type of fish [19]. The consumption of this species has its roots in the gastronomic tradition of salt cod or “Baccalà” and stockfish in Italy. In recent years, it has evolved to adapt to modern habits. Baccalà is made from the cod species *Gadus morhua* or *Gadus macrocephalus* through a process of salting and curing. Stockfish, originally from Norway, refers to unsalted cod that has been dried in the open air on wooden racks in Norway [20]. However, products commonly found on the market may come from different genera of fish (such as *Merluccius*, *Gadus*, *Theragra* and *Pollachius*), which are commercially known as ‘cod’, but which have different biological characteristics. These species may also differ significantly in terms of ecology, diet, metabolism, habitat and bioaccumulation capacity. Processed cod products are a constant presence in Italian supermarkets today, indicating an appreciation that transcends seasonality and encompasses all consumer segments. Given the high demand for processed fish products, it is important to assess the associated risks for consumers. However, the majority of studies in the literature focus on species such as salmon, tuna and mackerel, leaving important knowledge gaps regarding cod-based products. To this end, a study was conducted on various cod products (fresh and processed) to analyse their content of eight biogenic amines (cadaverine, histamine, 2-phenylethylamine, putrescine, tryptamine, tyramine, spermidine, and spermine) and twenty inorganic elements (including essential macro- and microelements, as well as toxic and potentially toxic elements). This approach enabled us to evaluate the quality of the analysed products in terms of essential inorganic elements and assess their safety by testing for the presence of biogenic amines and toxic and potentially toxic elements. The biogenic amines, in particular histamine, can cause ‘mackerel poisoning’ in consumers when present in high concentrations and are also indicators of freshness. Furthermore, given the high consumption of these products, this study also aimed to assess the intake of inorganic elements following ingestion of a certain amount of cod. The study aims to strengthen the current understanding of the safety of commercial cod products and support more informed consumer protection strategies.

## 2. Materials and Methods

### 2.1. Samples

Table 1 lists the cod-based products studied. Most of the samples come mainly from areas outside the Mediterranean, specifically from the Atlantic and Pacific oceans. Only one sample comes from the Mediterranean. All of the samples were purchased in 2025 from local market and supermarket in Messina, Italy.

The selection of the ten products was guided by the aim of providing a representative and realistic overview of the products available to consumers. Samples were chosen based on the following criteria: market representativeness; diversity of product types (fresh or processed); differences in manufacturing processes; declared geographical origin and species; and accessibility and actual consumption.

Three analytical replicates were performed for each cod sample after homogenization.

Table 1 illustrates the wide taxonomic variety: *Merluccius polli*, *Merluccius capensis*, *Merluccius paradoxus*, *Merluccius hubbsi*, *Merluccius productus*, *Merluccius merluccius*, all species of the genus *Merluccius*. In addition to these are: *Theragra chalcogramma* (Alaska pollock), *Pollachius virens* (saithe) and *Gadus morhua* (Atlantic cod). This diversity reflects the availability of species in the fishing grounds and the commercial choices of companies.

Where possible, the type of fishing used was also reported. Trawl nets are the most common method. For traditional products (baccalà and stockfish), however, the use of fishing hooks/longlines is recommended as this is considered a more selective technique. For the other samples, however, no details are provided.

### 2.2. Biogenic Amines Determination

Determining the biogenic amine content (cadaverine, histamine, 2-phenylethylamine, putrescine, tryptamine, tyramine, spermidine, and spermine) of cod samples involved three steps: extraction, derivatization and chromatographic analysis. This analysis was performed in accordance with the method described by Chiofalo et al. (2025) [21], with some modifications.

#### 2.2.1. Extraction

Two grams of muscle tissue were homogenised with 15 mL of 0.4 mol/L perchloric acid (Sigma-Aldrich, Steinheim, Germany), after which the mixture was centrifuged at 33.33 Hertz for 10 min at 4 °C. This step was repeated twice. The two resulting supernatants were then combined and centrifuged again at 41.67 Hertz for 5 min at 4 °C.

#### 2.2.2. Derivatization

A 1 mL aliquot of the extract was added to 250 µL of the internal standard 1,7-diaminoheptane (Sigma-Aldrich, Steinheim, Germany), 1 mL of saturated sodium bicarbonate solution (Sigma-Aldrich, Steinheim, Germany), 450 µL of 2 M sodium hydroxide (Sigma-Aldrich, Steinheim, Germany) and 500 µL of dansyl chloride (10 g/L, Merck, Darmstadt, Germany) to act as the derivatizing agent. The mixture was stirred for one minute and then incubated at 60 °C for thirty minutes to facilitate the derivatization reaction. Once complete, 1.7 mL of acetonitrile HPLC grade (Merck, Darmstadt, Germany) was added, and the mixture was centrifuged at 66.67 Hertz for 10 min to observe the separation of the two phases. The resulting supernatant was filtered through 0.22 µm filters and stored in the dark until chromatographic analysis, to avoid photoinduced degradation of the dansylate derivatives.

#### 2.2.3. Chromatographic Analysis

Biogenic amines were quantified using high-performance liquid chromatography (HPLC, Shimadzu Italia, Milan, Italy), which was equipped with an RF-20A fluorimetric detector (wavelengths: λ_ex_ = 320 nm, λ_em_ = 523 nm), a DGU-20A5R degasser, an LC-20AD pump and a CTO-20A column thermostat. Separation was performed using a SUPELCOSIL™ LC-18 column (25 cm × 4.6 mm, 5 µm) (Supelco, Bellefonte, PA, USA) at a temperature of 35 °C. The mobile phase consisted of HPLC-grade water (solvent A) and acetonitrile (solvent B) at a constant flow rate of 0.8 mL/min. The entire chromatographic analysis took 35 min.

### 2.3. Inorganic Elements Determination

Cod samples were collected, cleaned and sectioned in the laboratory. The muscle portions intended for analysis were homogenised and divided into aliquots for two different procedures: mercury determination using a direct analyser, and determination of the mineral content via microwave digestion followed by Inductively Coupled Plasma Mass Spectrometry (ICP-MS) and Inductively Coupled Plasma—Optical Emission Spectroscopy (ICP-OES) analysis.

#### 2.3.1. Mercury Determination by DMA-80

The total mercury concentration in cod muscle tissue samples was determined using a DMA-80 direct mercury analyser (Milestone, Bergamo, Italy), in accordance with the operating parameters reported by Bruno et al. (2024) [22], and with some modifications. Approximately 0.1 g of each sample was transferred directly into nickel boats. Each sample was subjected to a three-stage thermal programme: drying: 250 °C for 60 s; thermal decomposition: 750 °C for 150 s in an oxygen atmosphere, and catalysis and amalgamation. The released mercury was subsequently quantified by atomic absorption spectroscopy at 254 nm. A calibration curve was constructed using certified mercury standard solution (1000 mg/L, Analytika, Prague, Czech Republic). From this, seven standard solutions (1–100 μg/L) were prepared.

#### 2.3.2. Acid Digestion and ICP-MS and ICP-OES Analysis

To determine the mineral content, 0.5 g of each sample was subjected to acid digestion using a Milestone ETHOS 1 microwave mineraliser (Milestone, Bergamo, Italy) with PTFE vessels that are resistant to high temperatures and acid reagents. Each sample was treated with 8 mL of 69% *v*/*v* HNO_3_ and 2 mL of 30% *v*/*v* H_2_O_2_.

According to our previous study [7], the samples were then subjected to an acid digestion process consisting of five steps: (1) 10 min at a temperature ranging from 0 to 200 °C; (2) 5 min at a constant temperature of 200 °C; (3) 5 min with a temperature increase from 200 to 220 °C; (4) 5 min at a constant temperature of 220 °C; and (5) a 20 min cooling phase. After cooling, the samples were diluted with ultrapure water, filtered using 0.45 µm filters and a syringe, and stored at 4 °C until analysis. The certified matrix (ERM-CE278k-mussel tissue) and the blank solution were prepared under the same operating conditions as those used for the cod samples.

The inorganic elements analysed were divided into two groups. The first group, consisting of seven elements (Ca, Fe, K, Mg, Na, P, and Zn), was analysed using an ICP-OES ULTIMA 2 (HORIBA, Kyoto, Japan). The second group, composed of thirteen elements (Li, Be, Cr, Mn, Ni, Cu, As, Se, Mo, Cd, Sn, Sb, and Pb), was evaluated using an iCAP-Q ICP-MS (Thermo Scientific, Waltham, MA, USA). ICP-OES analysis was performed under the operating conditions reported by Potortì et al. (2026) [23]. Specifically, the following wavelengths were used: Ca (393.366 nm), Fe (259.940 nm), K (766.490 nm), Mg (279.553 nm), Na (588.995 nm), Zn (213.856 nm) and P (213.618 nm). On the other hand, the operating conditions used for the ICP-MS analysis were the same as those reported by Benameur et al. (2026) [24]. These conditions allowed the following isotopes to be monitored: ^7^Li, ^9^Be, ^52^Cr, ^55^Mn, ^60^Ni, ^63^Cu, ^75^As, ^80^Se, ^98^Mo, ^111^Cd, ^120^Sn, ^121^Sb, ^208^Pb. All the chemicals required for the analysis were of a very high purity and were supplied by J.T. Baker (Milan, Italy). Single-element standard solutions of Mg, Ca, P, Mn, Fe, Cu, Zn, Cd, Cr, Li, Be, Na, Ni, Mo, K, Pb, As, Se, Sn and Sb (1000 mg/L in 2% HNO_3_) were procured from Fluka (Milan, Italy) to construct seven-point calibration curves for the ICP-MS (range: 0.5–50 µg/L) and ICP-OES (range: 0.5–50 mg/L) analyses.

### 2.4. Element Intake

A key objective of this study was to quantify the intake of inorganic elements following the consumption of the analysed cod samples. To this end, the reference Recommended Dietary Allowances (RDAs) established by the European Commission [25,26,27] were adopted to calculate the essential macro- and microelement intake. In parallel, the intake of toxic and potentially toxic elements was calculated considering the reference Tolerable Daily Intake (TDI), Tolerable Weekly Intake (TWI) and Benchmark Dose Lower Confidence Limit 10% (BMDL01) [28,29,30,31,32,33]. All reference values are shown in Table 2.

The percentage of intake was calculated using a hypothetical daily fish consumption of 100 g an assumed average consumer weight of 70 kg (for As, Cd, Hg, Ni, and Pb). The average concentrations (mg/kg) in the samples were then multiplied by the amount consumed (kg/fish/day) to calculate daily exposure (mg/day).

### 2.5. Statistical Analysis

For the statistical analysis, the SPSS 19.0 software package (SPSS Inc., Chicago, IL, USA) was used. The results of the experiment were always given as the average value, plus or minus the standard deviation, of three analytical replicates for each cod sample. Statistically significant differences between cod samples based on brand and, consequently, geographical origin were evaluated using one-way analysis of variance (ANOVA). The level of statistical significance was set at *p* < 0.05. We excluded from the statistical analysis any analytes that we did not quantify in at least 50% of the samples. Finally, a value of LOD/2 was assigned to concentrations below the LOQ in only a few samples.

In this study, statistical analysis was conducted exclusively for descriptive purposes. This is because the included products do not represent replicates of the same population, but rather are heterogeneous in terms of species, ingredients, origin and technological processes. This implies that ANOVA, treated as a comparison between independent groups, cannot be interpreted inferentially since the ‘product type’ factor incorporates uncontrollable confounding variables. Therefore, ANOVA was only used for a preliminary exploration of variability between commercial categories, without attributing causal significance to *p* values.

## 3. Results and Discussion

### 3.1. Methods Validation

The ICP-MS, ICP-OES, DMA-80 and HPLC-FLD methods were validated in terms of their linearity, sensitivity, accuracy and precision. All results are reported in Tables S1 and S2.

Six standard solutions of biogenic amines were prepared at concentrations of 0.2, 1.0, 5.0, 10.0, 25.0 and 50.0 mg/L. The R^2^ value was lowest for cadaverine (0.9989) and highest for spermidine (0.9998) (Table S1).

The linearity of the methods used for inorganic elements was evaluated using seven-point curves, whose standard concentration ranges are reported in Section 2.3.1 and Section 2.3.2, and the correlation coefficient R^2^. The R^2^ value was greater than 0.9985 for all elements (Table S2).

To evaluate analytical sensitivity, the limits of detection (LOD) and quantification (LOQ) for each analyte were calculated using the formulas 3.3σ/S and 10σ/S, respectively, where σ is the standard deviation of the mean obtained from ten blank measurements and S is the slope of the corresponding calibration curve. A LOQ of 0.4 mg/kg was determined for all amines analysed (see Table S1). The LOQ values for inorganic elements ranged from 0.003 mg/kg for Cd and Pb to 3.383 mg/kg for K (see Table S2)

For the HPLC-FLD analysis, spike solutions containing different concentrations of biogenic amines (1.0, 5.0, 10.0 mg/L) were prepared. The accuracy of the ICP-MS, ICP-OES and DMA-80 methods was evaluated using the certified ERM-CE278k-Mussel Tissue matrix (Merck, Darmstadt, Germany) (n = 6). Furthermore, in the case of inorganic elements, if one of the targeted analytes was absent from the reference material, it was supplemented with known concentrations of the missing element(s) (10 mg/kg for P; 2 mg/kg for Be, Li, Mo, Sb e Sn). The recovery percentage was then obtained by subtracting the experimental value from the reference value. Good recovery rates were generally obtained for both biogenic amines and inorganic elements, with values falling within the optimal range of 80–120% (see Tables S1 and S2).

To evaluate the precision of the methods, two parameters were determined: intraday precision, analysing the enriched samples and the certified matrix on the same day; interday precision, calculated over a period longer than a week. Results, expressed as relative standard deviation (RSD%), were less than 1.2% and 1.3%, respectively, for both amines and mineral elements (see Tables S1 and S2).

### 3.2. Biogenic Amines

Table 3 shows the concentrations (mg/kg) of cadaverine, histamine, 2-phenylethylamine, putrescine, tryptamine, tyramine, spermidine and spermine found in the analysed cod samples. All the biogenic amines were detected in all fish products. In general, the study showed that fish products, particularly processed ones, contained low and variable levels of biogenic amines. This suggests that the raw materials were of a high quality and that the processing practices were correct. However, it also emphasises the importance of ongoing monitoring to ensure consumer safety.

The content of each biogenic amine varied depending on the type of product analysed and, consequently, its freshness or the processing it had undergone (see the *p*-values for all biogenic amines in Table 3, which are all less than 0.05). Generally, processed products had a higher biogenic amine content than fresh products. This trend is consistent with findings reported in other studies [34]. This result could be due to the fact that technological processes such as marinating, fermentation and salting can lead to the formation of biogenic amines. The total biogenic amine content ranged from 12.5 ± 0.54 mg/kg (sample F8) to 165.11 ± 3.08 mg/kg (sample F4). Tyramine was the most abundant amine in samples F1, F2, F4, F5 and F7, whereas cadaverine predominated in the remaining samples.

It is crucial to evaluate the presence of biogenic amines. Although they play an important role in various bodily processes (such as cellular development), consuming foods high in these compounds can have dangerous consequences, including increased blood pressure, tachycardia, nausea, etc. [35,36]. Furthermore, interest in determining the levels of biogenic amines in fresh and processed foods is growing, not only because of their toxicity, but also because the presence of biogenic amines in food products is often considered an indicator of deterioration during storage or ripening, and therefore an indicator of quality [37].

Histamine is the only amine regulated by EU legislation due to its toxicity, which can cause scombroid syndrome/histamine poisoning [38]. It is a substance formed when certain types of fish are not refrigerated properly before cooking or processing. However, other factors can also influence the decarboxylase activity of amino acids, including temperature, pH and the redox potential of the medium. For instance, a low pH level prompts microorganisms to produce decarboxylases as a protective response to acidity, whereas decreased redox potential increases histamine formation [39]. Fish products containing high levels of histamine may not look or smell bad, but they can still have toxic effects. In humans, symptoms include a tingling or burning sensation in the mouth or throat, skin rashes, headaches and diarrhoea, and they usually appear within an hour of eating [40]. In this regard, EC Regulation No. 2073/2005 and EU Regulation No. 1019/2013 set maximum limits of 100–200 mg/kg for histamine in fresh fish, and of 200–400 mg/kg for fishery products that have undergone enzymatic maturation treatment in brine [41,42].

Possible causes of unacceptable levels of biogenic amines include the use of poor-quality fish as the raw material for canned fish products, and inadequate handling techniques during processing. For instance, bacterial populations, including histamine-producing bacteria, can increase during fish thawing and handling. This may also be due to poorly cleaned equipment being used at various stages of the process, such as scaling, decapitation, gutting and filleting [43].

However, the histamine content found in our study was always below the regulatory limit. Specifically, this amine’s level ranged from 0.79 ± 0.07 mg/kg in sample F8 to 24.52 ± 1.26 mg/kg in sample F7 (Table 3). Furthermore, the histamine content of processed samples was higher than that of fresh cod, demonstrating the influence of different handling techniques on histamine content during the processing of the raw material. Kocar et al. (2021) observed a similar result when determining the content of biogenic amines in fresh and processed fish samples, finding a higher histamine level in the latter [37]. Specifically, the histamine content of our fresh cod sample was the lowest (0.79 ± 0.07 mg/kg) (Table 3). This demonstrates the quality of the product caught. Furthermore, the result obtained in the present study was significantly lower than that found in other analysed fish species in other studies. For instance, El-Ghareeb et al. (2021) examined the biogenic amine content of various fish species (e.g., Nile tilapia, mullet, mackerel, herring, sardines and tuna) purchased from local markets in Egypt [44]. They reported histamine levels that were consistently higher than those observed in our study, ranging from 17.700 ± 0.506 to 217.900 ± 0.3176 mg/kg [44]. This difference is probably due to the varying amounts of free histidine found in different species of fish [44]. With regard to ready-to-eat products, however, the histamine content was lower than that reported by Weremfo et al. (2020) for canned tuna, sardines and mackerel samples (range: not detected—64.05 mg/kg) [45]. In this case, too, the possible explanation could be the different type of raw material used to prepare the canned products. It is no coincidence that tuna and mackerel are the two species most susceptible to histamine contamination, and therefore most likely to cause “histamine poisoning” [46,47].

In addition to histamine, biogenic amines such as putrescine and cadaverine were typically found in fish identified as being affected by histamine poisoning [48]. As there is evidence that biogenic amines such as putrescine and cadaverine increase histamine toxicity in humans by inhibiting histamine-metabolising enzymes (diamine oxidase and histamine methyltransferase), attention should also be paid to their content [43,49]. This increase in toxicity precisely occurs when cadaverine and putrescine are present at concentrations five times higher than those of histamine [49]. Furthermore, evaluating these two biogenic amines is important because they may act as precursors to carcinogenic N-nitrosamines when heated in the presence of nitrites/nitrates [50]. Once again, the fresh product had lower levels than the processed products, in accordance with other studies [37]. Specifically, the lowest concentrations of cadaverine and putrescine were found in sample F8 (3.57 ± 0.15 mg/kg and 2.28 ± 0.08 mg/kg), while the highest concentrations were found in sample F4 (19.73 ± 0.55 mg/kg and 11.67 ± 0.34 mg/kg) (Table 3). Our results were consistent with those of Weremfo et al. (2020), who found cadaverine and putrescine concentrations of 0–27.23 mg/kg and 0–18.74 mg/kg, respectively, in canned tuna, sardines, and mackerel [45]. This demonstrates the influence of the quality of the starting product on the presence of these amines, which are not eliminated by the different processing steps.

Having toxic effects for human health, it is very crucial to underline the presence of tyramine in fish products [43]. Furthermore, consuming foods that contain high levels of tyramine can trigger migraines in sensitive individuals [36]. Tyramine influences the release of catecholamines, which cause adverse effects such as respiratory problems, migraines, vomiting and nausea, an increased heart rate and blood pressure, and hypertensive crises. It can also trigger neurological conditions such as schizophrenia, Parkinson’s disease and Reye’s syndrome [51]. The sample of fresh cod had the lowest tyramine content (1.40 ± 0.18 mg/kg), while cod fillet sticks had the highest (36.32 ± 0.58 mg/kg) (Table 3). The concentration of this amine in the fresh product was consistent with the findings of Andersen et al. (2019), equating to an average value of 2.0 mg/kg [52]. The increased presence of this amine in processed products may be due to poor handling and hygiene during the processing stage [51]. Furthermore, the presence of other ingredients or the use of high processing temperatures can favour the formation of greater amounts of tyramine. As expected, tyramine levels were higher in processed cod than in fresh cod in our samples. This could be due to a higher temperature favouring the proteolytic and decarboxylase activities of microorganisms, resulting in increased tyramine concentrations [51].

Tryptamine has similar effects to tyramine, such as increased blood pressure, headaches, fever and sometimes vomiting. However, the dosage at which these effects occur has not yet been specified [53]. High tryptamine levels may have been related to use of poor-quality raw fish [43]. In our study, however, tryptamine levels ranged from 1.71 ± 0.16 mg/kg in sample F8 (fresh cod) to 30.89 ± 1.54 mg/kg in sample F4 (Table 3). For this amine, the way the product is handled is also an influential parameter. This behaviour was highlighted in a study by Kosma et al. (2021), who examined the effect of storage time on trout samples kept on ice [54]. Specifically, the tryptamine content varied from 0.205 ± 0.007 mg/kg at time zero to 25,210 ± 0.947 mg/kg after 15 days of storage on ice.

Spermidine and spermine occur naturally in animal tissues. However, these amines may also originate from bacteria [55]. Spermidine acts as a biological biomarker for assessing food quality [51]. Poor raw material quality and inadequate storage techniques likely contribute to increased microbial contamination and bacterial decarboxylase activity, resulting in higher spermidine levels in samples [51]. Precise control of freezing temperatures can significantly impact spermidine levels by curbing microbial and enzymatic activities. As expected, this study found that the spermidine content of processed samples was consistently higher than that of fresh cod, with concentrations ranging from 1.28 ± 0.15 mg/kg (F8) to 30.80 ± 0.61 mg/kg (F4) (Table 3). Overall, the level of spermidine was higher than the level of spermine in all cod samples analysed (Table 3). This result was consistent with those of other studies in the literature [37,44].

Finally, the levels of 2-phenylethylamine detected were generally low. Concentrations ranged from 0.42 ± 0.05 mg/kg in sample F8 to 5.31 ± 0.20 mg/kg in sample F4. These values are consistent with the findings of Shiono et al. (2021), who reported 2-phenylethylamine contents in various fish products ranging from below the limit of quantification up to 2.0 mg/kg [56].

Biogenic amines are also used as indicators of food quality, signalling the degree of freshness and/or deterioration, and are used in the control of food processing [57]. The quality index (QI) is calculated using the following formula reported by Zare et al. (2017) [58]:

$$QI=\frac{Histamine+Putrescine+Cadaverine}{1+Spermine+Spermidine}$$

In this formula, the reported concentrations of biogenic amines are expressed in milligrams per kilogram. QI values can be divided into three groups: values between 0 and 1 indicate good product quality, values between 1 and 10 indicate acceptable levels, and values above 10 indicate product decomposition [59,60]. All of the cod products analysed fell into the second group, with values ranging from 1.0 to 3.1 (see Table 3).

Overall, the levels of biogenic amines in the fish products (fresh and processed) analysed in this study were low. These results therefore indicate that the fish used for these products was of good quality and that they were manufactured according to good manufacturing practices. However, despite the low levels of biogenic amines and histamine found, continued monitoring of these products is necessary for several reasons. Firstly, histamine production can vary significantly in different parts of the fish [50]. Consequently, it cannot be guaranteed that the remaining fish used to prepare this ready-to-eat product will not contain higher levels of histamine. Furthermore, continuous screening for these compounds is necessary because they are not destroyed by processes such as cooking, freezing and canning once formed [50]. This is essential to ensure product quality and safeguard consumer health.

### 3.3. Inorganic Elements

The inorganic profile of the cod samples analysed was determined using ICP-MS, ICP-OES and DMA-80 analyses (Table 4). In general, the elemental content varied depending on the product type, as indicated by the *p*-values obtained, which were all lower than 0.05 (see Table 4). The analysis shows that the levels of macro- and micronutrients in cod products vary according to species, processing methods and geographical origin. Values generally comply with regulatory limits, with some exceptions, and processed products tend to have higher concentrations than fresh fish. Any instance where the concentration of a toxic element exceeded the regulatory limit was highlighted in red in Table 4.

Of the analysed macro-elements (Ca, K, Mg, Na and P), potassium was the most abundant in all cod samples except F10. Being salted cod or baccalà, F10 naturally contains sodium as the main macronutrient (see Table 4).

The calcium content varied significantly depending on the product type (*p*-value < 0.05, see Table 4). The highest values were obtained for the stockfish and baccalà samples (F9 and F10). This is likely due to the drying processes these products undergo, which remove water content, increasing its concentration effect [61]. However, drying does not increase the absolute calcium content but increases its concentration per unit weight. A similar result was obtained by Abbas et al. (2021), who found that the calcium content of grey mullet samples increased following dry salting processes [62]. Generally, however, our Ca concentrations ranged from 153.84 ± 3.18 mg/kg (F2) to 1438.53 ± 54.74 mg/kg (F10) (see Table 4). The calcium content of most of the processed samples was higher than that of fresh cod. This is probably due to the exogenous contributions, such as salt or processing surfaces or presence of additional ingredients added to the raw material during handling [61,63]. However, the calcium level obtained for the fresh cod sample was consistent with the range reported by Noreen et al. (2025): 160–200 mg/kg [64]. This result is significant given the important role calcium plays in the human body, particularly in bone health and various metabolic processes [65].

For K, almost all the processed samples showed a higher content than the fresh cod, except for F2. This result is probably since no other ingredients were added to sample F2, i.e., South African cod fillets. As expected, the result obtained for this sample (3045.85 ± 29.30 mg/kg) was like that for fresh cod (3305.02 ± 7.50 mg/kg). In this case, therefore, it appears that product handling did not affect the potassium content. Furthermore, the baccalà sample (F10) showed the highest potassium concentration, at 12,039.57 ± 340.05 mg/kg. Noreen et al. (2025) reported a range of potassium concentrations in cod of 4130–4150 mg/kg [64], which is slightly higher than that found in our fresh sample. However, Kiczorowska et al. (2019) found slightly lower levels of potassium (K) in fresh and smoked cod, at 2.97 ± 0.41 g/kg and 2.31 ± 0.28 g/kg respectively [66]. The geographical origin of the product probably influenced the concentration of this element.

Magnesium levels were similar across all samples, ranging from 204.03 ± 2.61 to 1337.45 ± 11.80 mg/kg. Only stockfish (F9) and cod (F10) exhibited higher concentrations. As in previous studies, products that had undergone manipulation had a higher Mg content than the fresh product. As might be expected, factors such as salting techniques, drying times and the composition of the salt or other added ingredients also influence magnesium content. Studies have shown that salt containing high levels of magnesium ions can be used in fish products to improve their texture and colour [67]. A similar trend was observed in a study by Kiczorowska et al. (2019), in which smoked cod samples exhibited higher Mg concentrations than the fresh product (0.34 ± 0.54 vs. 0.27 ± 0.35 g/kg) [66].

The sodium concentration varied significantly among the analysed cod samples, with sample F1 having the lowest concentration (270.38 ± 1.76 mg/kg) and sample F10 having the highest (60,586.75 ± 103.11 mg/kg). The latter result was higher than expected: salting is a technique whereby salt penetrates the flesh of the fish when it is immersed in a concentrated saline solution [68]. All the other processed products, except F1, had a higher sodium content than the fresh product. This is undoubtedly due to the salt added during processing, which increases the product’s shelf life [69]. Furthermore, the sodium level in our fresh cod sample (F8: 739.05 ± 4.93 mg/kg) was higher than that reported by Noreen et al. (2025) for cod (range: 540–600 mg/kg) [64]. This difference may be related to the habitat of the analysed fish species. However, the implications of high sodium intake for human health must be taken into account, including its adverse effects on blood pressure. High blood pressure is a major risk factor for cardiovascular disease, including stroke and coronary heart disease [70,71].

Fish is an important source of phosphorus [72]. In our study, significant concentrations of this element were also obtained. The highest content was observed in sample F10 (8476.73 ± 271.42 mg/kg), probably due to the salting and drying processes, in addition to the presence of phosphate-based stabilisers. Emmanuel et al. (2020) also observed an increase in the content of Oreochromis niloticus samples after they were dried at different temperatures [73]. The lowest content was observed in sample F2 (1182.71 ± 46.34 mg/kg). This result is broadly consistent with that reported by Noreen et al. (2025) (range: 2030–2100 mg/kg) [64]. Overall, all samples were found to be a good source of phosphorus. This is important because P is an essential element for the human body, required for processes such as ATP synthesis, signal transduction and bone mineralisation [23].

Of the trace elements, iron and zinc were the most abundant (Table 4). The lowest content of both was detected in sample F2 (Fe: 4.49 ± 0.28 mg/kg; Zn: 3.39 ± 0.21 mg/kg), while the highest concentrations were observed in samples F10 and F3 for iron (26.23 ± 1.65 mg/kg) and zinc (21.32 ± 0.83 mg/kg), respectively. The concentration of Fe and Zn in the fresh cod sample falls within the range reported by Noreen et al. (2025) (range: 4–6 mg/kg for iron, 5–8 mg/kg for zinc) [64]. Similar trends were observed for copper, with levels generally higher in processed products than in fresh cod. Nevertheless, the value for fresh cod (0.54 ± 0.10 mg/kg) was slightly above that reported in the literature (range: 0.1–0.3 mg/kg) [64]. The differences observed for Fe, Zn and Cu reflect the combined effects of technological processes, geographical origin and storage methods. For example, El-Shaer et al. (2022) found an increase in iron content when switching from fresh tuna and sardine samples to canned ones [74].

Similar trends were observed for Mn, Mo and Cr: the processed samples had higher concentrations of these elements than the fresh product, with some exceptions. No elevated values of manganese, molybdenum and chromium were found. Specifically, the content of manganese ranged from 0.10 ± 0.02 mg/kg in sample F2 to 0.50 ± 0.05 mg/kg in sample F10; the content of molybdenum varied from values below the LOQ in sample F3 to 0.15 ± 0.01 mg/kg in sample F10; finally, samples F2, F6 and F7 were characterised from the lowest concentration of chromium (<LOQ), sample F10 the highest (0.05 ± 0.01 mg/kg).

Good concentrations of selenium were found in the analysed cod samples. The lowest content was found in sample F6 (0.21 ± 0.02 mg/kg), and the highest in F10 (1.46 ± 0.14 mg/kg). Fresh sample F8 (0.36 ± 0.03 mg/kg) showed levels comparable to those reported by Noreen et al. (2025) (range: 0.32–0.35 mg/kg) [64]. Furthermore, the fresh cod samples had lower selenium levels than F5, F7, F9 and F10, which were characterised by the addition of other ingredients or drying and salting processes that could have increased the selenium content. This result is significant given the fundamental role of selenium in the human body [75].

Regulation (EC) 915/2023 sets maximum limits for certain toxic elements, such as mercury, cadmium, and lead, in the muscle tissue of various species of fish [76]. For cod, the maximum limits are 0.30 mg/kg for Hg, 0.050 mg/kg for Cd and 0.30 mg/kg for Pb.

The mercury content ranged from 0.01 ± 0.00 mg/kg in samples F1–F7 to 0.27 ± 0.02 mg/kg in sample F10. Therefore, in general, all samples showed Hg levels below the regulatory limit. However, while the results are encouraging, evaluating the Hg content (in both inorganic and methylmercury forms) is essential given the danger it poses to human health, including neurotoxicity, immunotoxicity, genotoxicity, developmental toxicity, cardiovascular toxicity and metabolic effects [77,78]. With the exception of samples F9 and F10, processed samples showed lower Hg levels than the fresh product. This may be because smaller species are generally used in processed products, which accumulate smaller amounts of this element [79,80]. However, in the case of samples F9 and F10, the species factor also had a significant impact in addition to the influence of the drying processes, considering that *Gadus morhua*, characterised by its larger size compared to the other species, can accumulate greater quantities of mercury. Of course, mercury content is also related to other factors, such as the fish’s habitat, species and trophic level [81]. For example, Nava et al. (2023) analysed several canned samples of different fish species and reported variable concentrations of mercury (Hg) depending on the species of fish [5]. Tuna was characterised by the highest range (9.25–290.21 μg/kg) [5]. Brodziak-Dopierała et al. (2023) also determined the mercury content in different fish species and, again, the highest level was observed for tuna samples, while lower values were found for cod (around 0.04 mg/kg) [82].

Cadmium was found to be below the limit of quantification (LOQ) in many samples (F1, F2, F6, F8). The remaining samples, however, showed levels ranging between 0.01 ± 0.00 mg/kg (F3, F4, F5, F7) and 0.06 ± 0.01 mg/kg (F10). Consequently, only cod (F10) was, albeit slightly, above the threshold reported by the European Regulation. In light of the toxic effects of cadmium on human health, such as renal and hepatic dysfunction, pulmonary oedema, testicular damage, osteomalacia, adrenal gland and haematopoietic system damage, and cytotoxicity, this finding should be taken into consideration [83]. In general, the literature reports that cadmium content in fish products is low. For instance, Mielcarek et al. (2022) analysed various fish species and reported a range of cadmium (Cd) content from not analysed (NA) to 13.1 ± 6.89 µg/kg [84]. Polak-Juszczak et al. (2021) also found low levels of cadmium in the three *Gadidae* species (*Gadus morhua callarias* L., *Pollachius virens* L., and *Gadus morhua morhua* L.) analysed in their study, at concentrations ranging from 0.003 ± 0.00 to 0.005 ± 0.00 mg/kg [85]. The higher concentrations found in our F9 and F10 samples could be due to the drying processes used for the product, as well as the addition of salt. Several studies have shown cadmium content in samples of edible salt [86,87]. Furthermore, the Cd content of our fresh cod sample was lower than that reported by Jarosz-Krzemińska et al. (2021) for a North-East Atlantic and Baltic Sea cod samples (below the LOQ vs. 0.15 ± 0.02 mg/kg vs. 0.21 ± 0.02 mg/kg) [88]. This is probably due to the products’ different origins and, consequently, the different levels of sea contamination.

It is also essential to determine the lead content in food matrices. Exposure to lead can cause various toxic effects in humans. These include cardiovascular, haematological, neurological, nephrotoxic, reproductive, bone and cellular toxicity [89]. However, all samples had concentrations well below the regulatory threshold (0.30 mg/kg): the lead levels ranged from 0.01 ± 0.00 mg/kg in sample F8 to 0.08 ± 0.01 mg/kg in sample F10. Once again, the difference between the analysed samples is due to how they were handled and whether any ingredients were added that could increase the presence of this toxic element. Furthermore, the geographical origin of the raw material also plays a fundamental role in the case of lead. As expected, the lead concentration found in our fresh sample (F8) differed from that reported by Jarosz-Krzemińska et al. (2021) (below detection limit for Atlantic cod and 1.15 ± 0.14 mg/kg for Baltic cod) [88].

The levels of arsenic vary, ranging from a minimum of 0.15 ± 0.04 mg/kg (F6) to a maximum of 0.80 ± 0.09 mg/kg (F10). Regulation (EU) 2025/1891 sets the maximum level of inorganic arsenic in cod at 0.10 mg/kg [90]. Consequently, arsenic content exceeding this regulatory limit was found in all cod samples analysed. These findings should serve as a wake-up call, given the various harmful consequences of arsenic exposure. This element affects people of all genders and ages. Its toxic effects include gastrointestinal disorders, liver and pancreatic disease, cardiovascular disease, kidney disease, and reproductive disorders [91]. However, most of the arsenic found in fish products is organic (arsenobetaine), and this is considered less toxic than the inorganic form [92]. The chemistry of arsenic is highly complex, and its speciation influences its bioavailability and bioaccumulation in marine organisms. The bioavailability of inorganic and organic arsenic in marine fish may be influenced by the relative proportions of these compounds. This could explain differences in bioaccumulation [93]. Arsenic, once absorbed by aquatic organisms and accumulated in their bodies, occurs primarily in the form of organic compounds, with a low percentage accumulating in the form of the more toxic inorganic arsenic. The accumulation of different forms of arsenic depends primarily on the species and tissue analysed [94]. Inorganic arsenic is the most toxically relevant form, as it is associated with well-documented adverse effects, such as carcinogenicity (skin, lung, bladder), skin lesions, neurotoxicity, cardiovascular changes, and metabolic disorders. Organic species, on the other hand, present a much more heterogeneous toxicity: arsenobetaine is considered to be of low risk and is rapidly excreted, while arsenosugars and methylated metabolites (monomethylarsonic acid (MMA) and dimethylarsinic acid (DMA)) have a less defined profile and show experimental evidence of potential cytotoxicity and, in the case of DMA, of carcinogenicity in animal models [95].

Therefore, as our study determined the total arsenic content (i.e., the sum of the inorganic and organic forms), we cannot state with certainty whether the regulated inorganic arsenic content has been exceeded. Clearly, the accumulation of this element is related to its presence in the fish species’ environment [96,97].

Tin is also regulated at a European level [76]. However, maximum limits are reported for canned products. Consequently, these limits cannot be applied to the samples analysed in this study. The levels found were generally low, ranging from below the LOQ (F3, F4, F5 and F7) to 0.17 ± 0.02 mg/kg (F10).

The concentrations of nickel, antimony, lithium and beryllium were all below their respective limits of quantification in almost all of the samples analysed.

### 3.4. Element Intake

In addition to analysing the levels of biogenic amines and inorganic elements in samples of fresh and processed cod, this study aimed to determine the intake of inorganic elements when eating a specific quantity of this type of fish. Given that these products are often already portioned, an average consumption of 100 g of cod per day was used for this calculation. Table 5 shows the percentages of intake for each inorganic element. Elements for which concentrations were consistently below the LOQ (Be, Li, Sb), or those for which there are no reference values, are not included in this table.

As shown in Table 5, consuming 100 g of cod per day results in variable intake of inorganic elements. This variation appears to be related to the type of element and cod product analysed. The highest intake percentages for macronutrients were observed for potassium, with values between 15% (F2) and 60% (F10), indicating that cod is a good source of this element. Good intake percentages were also obtained for sodium and phosphorus. Specifically, the lowest value for sodium was shown by the F1 sample (2%), while the highest value was shown by the F10 cod sample (>100%). This is an expected result, given the salting process to which this product is subjected. For phosphorus, the lowest percentages were shown by sample F2 (17%), while sample F10 exceeded the daily intake, showing percentages higher than 100%. Finally, calcium and magnesium intake percentages were lower than those of the other macronutrients (Ca: 2–18%, Mg: 5–36%).

Trace element intake was also quite variable. The percentages obtained for iron, zinc, manganese, copper, molybdenum and chromium were always 30% or less. Selenium had the highest range, with percentages consistently above 38%. Samples F7, F9 and F10 had even higher percentages, exceeding 100%. This shows that cod can be a good source of selenium. However, while selenium has significant health benefits (for the brain, cardiovascular system, etc.) [98], it should be noted that taking more than the recommended daily amount can cause toxicity (selenosis) [99].

The intake of most toxic and potentially toxic elements (Cd, Hg, Ni and Pb) was consistently below 24%. However, concerning values were obtained for arsenic, with levels exceeding 100% in almost all samples. As noted in the previous paragraph, it should be noted that this study calculated total arsenic content (the sum of inorganic and organic forms). Furthermore, the TDI used for the calculation only refers to inorganic arsenic. Therefore, although this value is concerning, it must be weighed against the two aforementioned variables.

Consequently, given the variable risks associated with consuming the various inorganic elements analysed, it may be advisable to reduce the daily amount of cod consumed in order to minimise the intake of components that exceed the daily requirement. In this regard, if we consider the global daily consumption of demersal fish reported by Food and Agriculture Organization Corporate Statistical Database (FAOSTAT) (approximately 7 g per day) [100], which includes cod, all intake percentages, especially those of toxic and potentially toxic elements, would fall within the required range. However, it should also be noted that the intake of inorganic elements varies depending on the lifestyles of different countries, which obviously consume different quantities of fish. FAOSTAT indicates a daily consumption of 21 g of demersal fish in Italy, for example [100]. Given this figure, all intake percentages are below 100%.

### 3.5. Study Limitations

Due to the combination of taxonomic heterogeneity, a lack of biological replicates and a small sample size, the results must be interpreted with caution, which limits the scope of the study. As an exploratory and descriptive study, it provides a preliminary overview of the compositional variability of commercial products labelled ‘cod’ but does not allow for generalizable conclusions or rigorous comparisons between species or product categories. Future studies should include larger samples, biological replicates and systematic distinctions between species and technological processes.

## 4. Conclusions

The investigation of cod-based fish products revealed generally satisfactory quality. Freshness indicators were moderate and histamine levels were consistently below regulatory limits. However, the accumulation of biogenic amines is influenced by processing and storage conditions and is higher in processed products than in fresh ones.

The content of macro- and microelements varies depending on the product type and cod species/origin. Overall, these foods are a good source of calcium, potassium, magnesium, phosphorus and sodium, and provide a moderate amount of iron, zinc, copper and selenium, confirming their nutritional value.

Only one sample exceeded the European limit for cadmium, while all products showed total arsenic concentrations above the reference value. As the inorganic fraction was not separated, further speciation-specific investigations are required. Furthermore, consuming standard portions (100 g) can lead to high intakes of Na, P, Se and, in particular, As, suggesting that caution should be exercised regarding consumption quantities to avoid potential toxicological risks.

Due to the widespread availability of these products, continuous monitoring along the supply chain is essential to prevent exposure to contaminants and ensure consistent quality and traceability standards. Future studies should include larger sample sizes, biological replicates and systematic distinctions between species and technological processes to improve the robustness of risk assessments and understanding of observed variability.

## Figures and Tables

**Table 1:** Sample information (type, declared species, FAO zone, fishing type, and ingredients) reported in the label.

Sample	Declared Species	FAO Zone	Fishing Type	Ingredients
Decapitated cod (F1)	*Merluccius polli*	34 (Central-Eastern Atlantic)	Trawl nets	Cod
South African cod fillets (F2)	*Merluccius capensis* and *Merluccius paradoxus*	47 (Southeastern Atlantic)	no data	Cod. May contain molluscs and crustaceans.
Cod burger (F3)	*Theragra chalcogramma*	61 (Alaska pollock)	no data	Cod, potato starch, corn flour, water, sunflower oil, wheat flour, salt, parsley, caramelised sugar, yeast. This product may contain traces of soy, mustard, molluscs and crustaceans.
Cod fillet sticks (F4)	*Theragra chalcogramma*	67–71 (Northeastern and central-western Pacific)	no data	Cod, breadcrumbs, yeast, salt, paprika, turmeric, water, sunflower oil, wheat flour, salt.
Cod fillet sticks (F5)	*Merluccius productus*	DP 67 (Northeast Pacific)	no data	Cod, wheat flour, rapeseed oil, water, salt, natural yeast, paprika, potato starch, turmeric. May contain traces of mustard.
Frozen Atlantic cod fillet medallions (F6)	*Merluccius hubbsi*	41 (Southwestern Atlantic)	Trawl nets	Cod. May contain molluscs and crustaceans.
Golden Norwegian cod fillets with a thin breadcrumb Coating (F7)	*Pollachius virens*	27 (Northeast Atlantic)	Caught	Cod, carbonate, breadcrumbs, wheat flour, sunflower oil, water, potato starch, salt, rice flour, black pepper. May contain traces of crustaceans, eggs, soy, milk, mustard, molluscs, and celery.
Fresh cod (F8)	*Merluccius merluccius*	37.2.2 (Central Mediterranean, Ionian Sea)	Trawl nets	Cod.
Stockfish (F9)	*Gadus morhua*	27.2.A2 (Northeast Atlantic, Norwegian Sea)	Fishing hooks/ Longlines	Cod, water.
Baccalà (F10)	*Gadus morhua*	27.2.A2 (Northeast Atlantic, Norwegian Sea)	Fishing hooks/ Longlines	Cod, salt, stabilisers (E451).

**Table 2:** Reference RDAs for essential macro- and micronutrients, and TDI, TWI and BMDL01 for toxic and potentially toxic elements.

Element	RDA (mg/Day)	TDI (μg/kg_b.w._/Day)	TWI (μg/kg_b.w._/Week)	BMDL01 (μg/kg_b.w._/Day)
As		0.3		
Cd			2.5	
Ca	800			
Cr	0.040			
Cu	1			
Fe	14			
Hg		4		
K	2000			
Li	1			
Mg	375			
Mn	2			
Mo	0.050			
Na	1500			
Ni		22		
P	700			
Pb				0.5
Se	0.055			
Zn	10			

**Table 3:** Content of biogenic amines (mg/kg) in fresh and processed cod. The results are shown as the average value, with the standard deviation of the three replicates. *p*-values in bold are statistically significant according to one-way ANOVA (*p* < 0.05).

Biogenic Amine	F1	F2	F3	F4	F5	F6	F7	F8	F9	F10	*p*-Value
Cadaverine	12.75 ± 0.35	14.14 ± 0.47	18.53 ± 0.38	19.73 ± 0.55	11.99 ± 0.38	19.35 ± 0.23	15.47 ± 0.41	3.57 ± 0.15	10.03 ± 0.62	8.92 ± 0.18	**<0.05**
Histamine	7.17 ± 0.59	14.47 ± 0.78	11.02 ± 0.43	20.17 ± 0.64	8.28 ± 0.54	17.25 ± 0.31	24.52 ± 1.26	0.79 ± 0.07	2.25 ± 0.16	3.74 ± 0.16	**<0.05**
2-Phenylethylamine	1.10 ± 0.08	3.24 ± 0.08	1.19 ± 0.06	5.31 ± 0.20	4.25 ± 0.09	0.94 ± 0.04	4.65 ± 0.21	0.42 ± 0.05	0.68 ± 0.03	0.49 ± 0.05	**<0.05**
Putrescine	5.11 ± 0.10	9.56 ± 0.33	4.72 ± 0.17	11.67 ± 0.34	8.51 ± 0.33	7.76 ± 0.19	10.51 ± 0.23	2.28 ± 0.08	3.39 ± 0.15	4.08 ± 0.18	**<0.05**
Tryptamine	13.15 ± 0.45	23.37 ± 0.56	7.52 ± 0.28	30.89 ± 1.54	24.75 ± 0.79	6.63 ± 0.28	27.04 ± 0.50	1.71 ± 0.16	2.72 ± 0.18	2.30 ± 0.15	**<0.05**
Tyramine	17.55 ± 0.57	32.65 ± 0.46	11.19 ± 0.28	36.32 ± 0.58	27.07 ± 0.15	8.52 ± 0.28	31.48 ± 1.21	1.40 ± 0.18	2.59 ± 0.18	3.54 ± 0.26	**<0.05**
Spermidine	7.36 ± 0.45	15.21 ± 0.47	5.41 ± 0.21	30.80 ± 0.61	22.21 ± 1.35	6.58 ± 0.18	25.18 ± 0.33	1.28 ± 0.15	2.50 ± 0.07	2.86 ± 0.12	**<0.05**
Spermine	3.60 ± 0.33	6.39 ± 0.23	3.09 ± 0.05	10.22 ± 0.54	5.28 ± 0.26	3.35 ± 0.13	8.14 ± 0.40	1.05 ± 0.17	1.90 ± 0.05	2.48 ± 0.31	**<0.05**
Total biogenic amines	67.79 ± 1.15	119.03 ± 0.97	62.67 ± 0.95	165.11 ± 3.08	112.34 ± 2.94	70.38 ± 0.70	146.99 ± 2.99	12.5 ± 0.54	26.06 ± 1.14	28.41 ± 0.40	-
Quality index (QI)	1.7	1.7	2.5	1.2	1.0	3.1	1.5	1.0	3.1	2.6	-

**Table 4:** Content of inorganic elements (mg/kg) in fresh and processed cod. The results are shown as the average value, with the standard deviation of the three replicates. *p*-values in bold are statistically significant according to one-way ANOVA (*p* < 0.05). Values above the regulatory limit are displayed in red.

Element	F1	F2	F3	F4	F5	F6	F7	F8	F9	F10	*p*-Value
As	**0.24 ± 0.02**	**0.41 ± 0.03**	**0.57 ± 0.02**	**0.52 ± 0.03**	**0.52 ± 0.04**	**0.15 ± 0.04**	**0.25 ± 0.03**	**0.32 ± 0.02**	**0.59 ± 0.02**	**0.80 ± 0.09**	**<0.05**
Be	<LOQ	<LOQ	<LOQ	<LOQ	<LOQ	<LOQ	<LOQ	<LOQ	<LOQ	<LOQ	-
Ca	196.44 ± 8.36	153.84 ± 3.18	366.37 ± 6.21	264.64 ± 2.43	243.07 ± 2.82	176.79 ± 3.42	223.24 ± 2.86	167.02 ± 4.75	516.88 ± 3.49	1438.53 ± 54.74	**<0.05**
Cd	<LOQ	<LOQ	0.01 ± 0.00	0.01 ± 0.00	0.01 ± 0.00	<LOQ	0.01 ± 0.00	<LOQ	0.04 ± 0.01	**0.06 ± 0.01**	**<0.05**
Cr	0.01 ± 0.00	<LOQ	0.03 ± 0.01	0.04 ± 0.00	0.02 ± 0.01	<LOQ	<LOQ	0.01 ± 0.00	0.03 ± 0.01	0.05 ± 0.01	**<0.05**
Cu	0.58 ± 0.03	0.63 ± 0.03	0.81 ± 0.02	0.87 ± 0.03	0.91 ± 0.03	0.48 ± 0.03	1.02 ± 0.03	0.54 ± 0.10	1.15 ± 0.08	1.93 ± 0.07	**<0.05**
Fe	6.85 ± 0.34	4.49 ± 0.28	14.22 ± 0.33	11.47 ± 1.25	13.92 ± 0.84	5.10 ± 0.07	9.15 ± 0.28	5.41 ± 0.11	9.12 ± 0.51	26.23 ± 1.65	**<0.05**
Hg	0.01 ± 0.00	0.01 ± 0.00	0.01 ± 0.00	0.01 ± 0.00	0.01 ± 0.00	0.01 ± 0.00	0.01 ± 0.00	0.08 ± 0.03	0.14 ± 0.01	0.27 ± 0.02	**<0.05**
K	3535.88 ± 94.60	3045.85 ± 29.30	6657.77 ± 74.19	5024.40 ± 30.43	5387.94 ± 98.50	3362.81 ± 48.22	5656.27 ± 72.36	3305.02 ± 7.50	4671.38 ± 79.94	12,039.57 ± 340.05	**<0.05**
Li	<LOQ	<LOQ	<LOQ	<LOQ	<LOQ	<LOQ	<LOQ	<LOQ	<LOQ	<LOQ	-
Mg	278.07 ± 6.40	204.03 ± 2.61	249.61 ± 2.03	232.94 ± 3.49	250.27 ± 0.85	285.88 ± 3.41	310.92 ± 1.45	249.61 ± 6.64	531.02 ± 56.13	1337.45 ± 11.80	**<0.05**
Mn	0.19 ± 0.02	0.10 ± 0.02	0.38 ± 0.01	0.41 ± 0.02	0.43 ± 0.03	0.17 ± 0.02	0.24 ± 0.02	0.13 ± 0.02	0.32 ± 0.03	0.50 ± 0.05	**<0.05**
Mo	0.03 ± 0.01	0.02 ± 0.01	<LOQ	0.04 ± 0.01	0.02 ± 0.01	0.03 ± 0.00	0.05 ± 0.01	0.04 ± 0.01	0.08 ± 0.01	0.15 ± 0.01	**<0.05**
Na	270.38 ± 1.76	2404.26 ± 7.27	2643.15 ± 4.74	4441.36 ± 3.65	3453.89 ± 9.24	1517.59 ± 8.43	2439.90 ± 2.37	739.05 ± 4.93	10,206.49 ± 79.20	60,586.75 ± 103.11	**<0.05**
Ni	<LOQ	<LOQ	<LOQ	<LOQ	<LOQ	<LOQ	<LOQ	<LOQ	0.01 ± 0.00	0.02 ± 0.01	-
P	1415.00 ± 24.23	1182.71 ± 46.34	2163.14 ± 23.60	1982.31 ± 13.68	2041.93 ± 17.65	1318.11 ± 3.32	2035.45 ± 6.81	1304.03 ± 34.53	2182.38 ± 68.85	8476.73 ± 271.42	**<0.05**
Pb	0.02 ± 0.01	0.03 ± 0.01	0.05 ± 0.00	0.05 ± 0.01	0.05 ± 0.01	0.02 ± 0.01	0.04 ± 0.00	0.01 ± 0.00	0.07 ± 0.00	0.08 ± 0.01	**<0.05**
Sb	<LOQ	<LOQ	<LOQ	<LOQ	<LOQ	<LOQ	<LOQ	<LOQ	<LOQ	<LOQ	-
Se	0.32 ± 0.01	0.25 ± 0.03	0.26 ± 0.02	0.22 ± 0.01	0.44 ± 0.03	0.21 ± 0.02	0.65 ± 0.02	0.36 ± 0.03	0.84 ± 0.09	1.46 ± 0.14	**<0.05**
Sn	0.04 ± 0.01	0.02 ± 0.01	<LOQ	<LOQ	<LOQ	0.02 ± 0.01	<LOQ	0.05 ± 0.01	0.12 ± 0.01	0.17 ± 0.02	**<0.05**
Zn	5.04 ± 0.09	3.39 ± 0.21	21.32 ± 0.83	15.63 ± 0.75	19.25 ± 1.03	4.52 ± 0.08	11.75 ± 0.73	4.04 ± 0.16	8.10 ± 0.15	14.59 ± 0.93	**<0.05**

**Table 5:** Element intake (%) of inorganic elements from the consumption of 100 g/day of cod samples.

Element (%)	F1	F2	F3	F4	F5	F6	F7	F8	F9	F10
As	**>100**	**>100**	**>100**	**>100**	**>100**	71	**>100**	**>100**	**>100**	**>100**
Ca	2	2	5	3	3	2	3	2	6	18
Cd	-	-	4	4	4	-	4	-	16	24
Cr	3	-	8	10	5	-	-	3	8	13
Cu	6	6	8	9	9	5	10	5	12	19
Fe	5	3	10	8	10	4	7	4	7	19
Hg	<1	<1	<1	<1	<1	<1	<1	3	5	10
K	18	15	33	25	27	17	28	17	23	60
Mg	7	5	7	6	7	8	8	7	14	36
Mn	1	1	2	2	2	1	1	1	2	3
Mo	6	4	-	8	4	6	10	8	16	30
Na	2	16	18	30	23	10	16	5	68	**>100**
Ni	-	-	-	-	-	-	-	-	<1	<1
P	20	17	31	28	29	19	29	19	31	**>100**
Pb	6	9	14	14	14	6	11	3	20	23
Se	58	45	47	40	80	38	**>100**	65	**>100**	**>100**
Zn	5	3	21	16	19	5	12	4	8	15

“**>100**”: intake exceeding the RDA or TDI. “-”: the percentage intake could not be calculated because the concentrations were below the LOQ.

## Data Availability

The original contributions presented in this study are included in the article/Supplementary Materials. Further inquiries can be directed to the corresponding author.

## Supplementary Materials

The following supporting information can be downloaded at: https://www.mdpi.com/article/10.3390/foods15071202/s1, Table S1. HPLC-FLD validation parameters; Table S2. ICP-MS, ICP-OES, and DMA-80 validation parameters.
