# Supplementary material for: Safety of Commercial Cod Products and Potential Impact on Consumers
Source: Foods. 2026 Apr 2;15(7):1202. doi: 10.3390/foods15071202 (PMC13072758; doi:10.3390/foods15071202)
Supplement: Supplementary file 1 [file foods-15-01202-s001.zip › foods-4201232-supplementary.pdf]

Table S1. HPLC-FLD validation parameters.

	R^2	LOD (mg/kg)	LOQ (mg/kg)	Recovery (%)	Precision (RSD%)	
					Intraday	Interday
Cadaverine	0.9989	0.12	0.40	91.50	1.2	1.3
Histamine	0.9991	0.12	0.40	96.00	1.1	1.2
2-Phenylethylamine	0.9997	0.12	0.40	98.75	1.0	1.2
Putrescine	0.9990	0.12	0.40	95.40	1.1	1.3
Tryptamine	0.9992	0.12	0.40	96.50	0.8	1.0
Tyramine	0.9993	0.12	0.40	97.25	0.9	1.0
Spermidine	0.9998	0.12	0.40	98.50	1.0	1.1
Spermine	0.9985	0.12	0.40	90.55	1.0	1.2

Table S2. ICP-MS, ICP-OES, and DMA-80 validation parameters.

	R^2	LOD (mg/kg)	LOQ (mg/kg)	Recovery (%)	Precision (RSD%)	
					**Intraday**	**Interday**
As	0.9997	0.003	0.010	98.50	1.2	1.3
Be**	0.9993	0.003	0.010	95.25	1.1	1.2
Ca	0.9990	0.688	2.270	93.50	1.2	1.3
Cd	0.9999	0.001	0.003	101.50	0.9	1.0
Cr	0.9996	0.003	0.010	97.00	1.0	1.1
Cu	0.9997	0.008	0.026	97.50	0.9	1.0
Fe	0.9994	0.575	1.898	96.95	0.8	0.9
Hg	0.9998	0.003	0.010	96.75	0.9	1.0
K	0.9985	1.025	3.383	90.55	1.2	1.3
Li**	0.9993	0.003	0.010	95.90	1.2	1.3
Mg	0.9992	0.665	2.195	96.00	1.1	1.2
Mn	0.9995	0.008	0.026	95.25	1.0	1.1
Mo**	0.9995	0.004	0.013	98.20	0.8	1.0
Na	0.9989	0.895	2.954	91.15	1.2	1.3
Ni	0.9998	0.003	0.010	96.80	0.8	1.0
P*	0.9986	0.995	3.284	94.23	1.2	1.3
Pb	0.9997	0.001	0.003	101.00	0.8	0.9
Sb**	0.9993	0.003	0.010	96.55	1.2	1.3
Se	0.9994	0.003	0.010	97.55	0.8	1.0
Sn**	0.9995	0.003	0.010	95.75	1.2	1.3
Zn	0.9994	0.538	1.775	97.25	1.1	1.2

*The recovery of this element was tested by surrogate spike at 10.00 mg/kg.

** The recovery of these elements was tested by surrogate spikes at 2.00 mg/kg.
